# Effects of Mo alloying on stability and diffusion of hydrogen in the Nb_16_H phase: a first-principles investigation

**DOI:** 10.1039/c9ra03401c

**Published:** 2019-06-21

**Authors:** Dianhui Wang, Yang Wu, Zhenzhen Wan, Feng Wang, Zhongmin Wang, Chaohao Hu, Xiaotian Wang, Huaiying Zhou

**Affiliations:** School of Materials Science and Engineering, Guilin University of Electronic Technology Guilin 541004 P. R. China zmwang@guet.edu.cn chaohao.hu@guet.edu.cn; Guangxi Key Laboratory of Information Materials, Guilin University of Electronic Technology Guilin 541004 P. R. China; School of Physical Science and Technology, Southwest University Chongqing 400715 P. R. China xiaotianwang@swu.edu.cn

## Abstract

First-principles calculations and the method of climbing-image nudged elastic band were used to investigate the effects of Mo alloying on the structural stability, mechanical properties, and hydrogen-diffusion behavior in the Nb_16_H phase. The Nb_12_Mo_4_H phase (26.5 at% Mo) was found to be the most thermodynamically stable structure, with a low Δ*H*_f_ value (−0.26 eV) and high elastic modulus. Calculations revealed that the tetrahedral interstitial site (TIS) was the predominant location of H in both Nb_16_H and Nb_12_Mo_4_H phases. The calculated H-diffusion energy barrier and the diffusion coefficient of the Nb_12_Mo_4_H phase were 0.153 eV and 5.65 × 10^−6^ cm^2^ s^−1^ (300 K), respectively, which suggest that the addition of Mo would lead to a lower energy barrier and high diffusion coefficients for the Nb_16_H phase, thus improving the hydrogen-permeation properties of Nb metal.

## Abstract 2

First-principles calculations and climbing-image nudged elastic band method were used to investigate the effects of Mo alloying on the structural stability, mechanical properties, and hydrogen-diffusion behavior of Nb.

## Graphical abstract

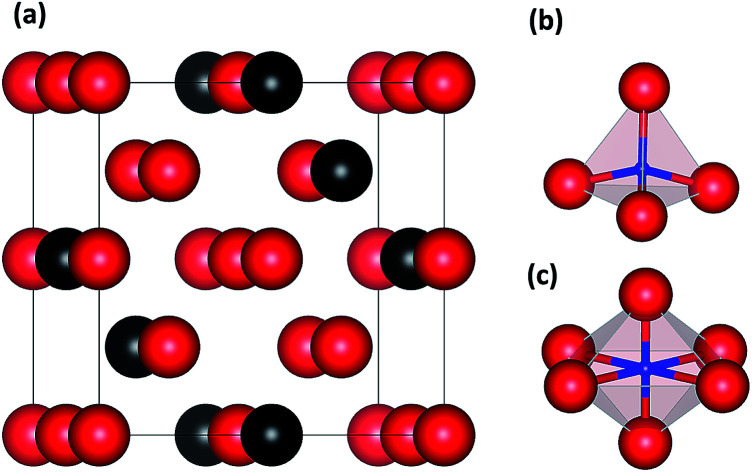

## 1. Introduction

Hydrogen is not only an important raw material for chemical and petrochemical industries, but also a potential clean fuel as well as a good energy carrier. Pure hydrogen does not exist as a natural resource like coal and oil, however. Since it has to be produced from hydrogen-containing compounds, a safe, low-cost, and highly efficient separation and purification technology is always required. Hydrogen-permeable alloy membranes have been well regarded as the most important materials for hydrogen separation and purification.^1–5^ Currently, group V metals (vanadium, niobium, and tantalum) have attracted many investigations as promising hydrogen-separation materials owing to their lower price and higher hydrogen permeability than those of currently used Pd-based alloys.^3,6^ However, there is still a large barrier to practical application of these metals because of their poor resistance to hydrogen embrittlement.^3,7,8^ Experimental studies have verified that alloying the metals is an effective way to solve this problem.^6–9^

Niobium is one of the most promising hydrogen-permeable candidates for membranes because it possesses a good combination of excellent high-temperature mechanical properties and corrosion resistance.^10–12^ Recent theoretical research performed by Watanabe *et al.* revealed that the addition of W could decrease the hydrogen solubility in Nb and therefore improve its resistance to hydrogen embrittlement.^6,13^ Hu *et al.* performed a similar study and found that the addition of W can improve the mechanical properties of the Nb_16_H phase, decrease the structural stability of the Nb_15_WH (tetrahedral (T)) phase, lower the diffusion barrier of H, and enhance diffusion paths for H.^14^ Both W and Mo are high-*Z* refractory metals (*i.e.*, refractory metals containing impurities with high atomic numbers (*Z*)) with similar physical properties. Moreover, Mo has several characteristic properties: compared with W, Mo has a lower melting point (2883 K) and a lower erosion rate, while H has higher diffusivity and lower solubility in Mo, leading to lower H retention.^15–17^ These characteristics make Mo an important alloying candidate for Nb-based alloy membranes for hydrogen permeation. However, since relevant works have not been reported in the literature, it is necessary to engage in first-principles theoretical investigations that are free from any experimental limitations on the effect of Mo addition on the structure and diffusion properties of the NbH phase in a first-principles way. Such calculations will also contribute to the understanding and design of H-storage and H-separation materials based on Nb.

The effects of the addition of Mo to the electronic structure, structural stability, H diffusion, and mechanical properties of the NbH phase were investigated by first-principles calculation based on density functional theory. Nb_16_H was purposely selected and four Mo atoms were added to reach the equivalent of an experimental composition of 25 at% Mo in NbH.^6,7,13,18^ The calculated results revealed that such addition would lead to a lower energy barrier of H diffusion, a higher diffusion coefficient, and improved mechanical properties.

## 2. Computational method

The calculations were carried out by using the Vienna Ab-initio Simulation Package (VASP).^19^ The interactions between core and valence electrons were described with the projector augmented wave (PAW).^20^ The exchange and correlation functions were generalized gradient approximations (GGAs) developed by Perdew *et al.*^21^ An energy cutoff of 360 eV was used for the plane-wave basis sets, and the *K* points set used in our calculations is 5 × 5 × 5 grid generated by Monkhorst–Pack schema.^22^ During structure relaxation of the lattice parameters, the volume and atomic positions were fully optimized with in-symmetry restrictions until the total energy converged to 10^−5^ eV in the self-consistent loop, and the criterion of force used in the calculations is 0.01 eV Å^−1^ atom^−1^.

**Fig. 1:**
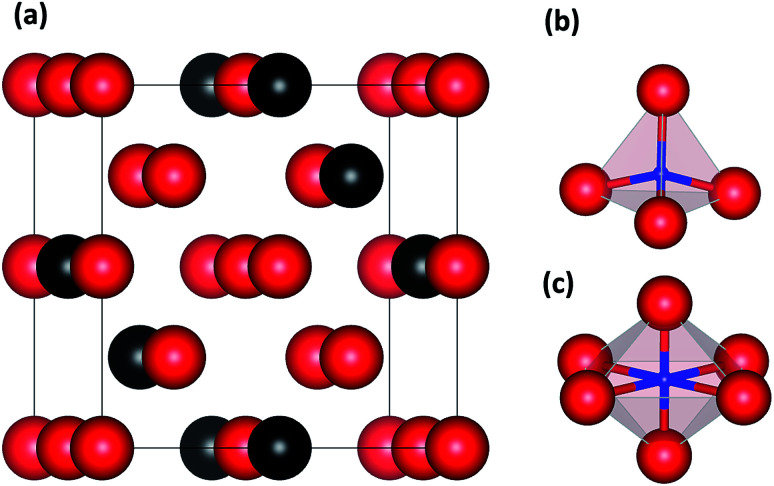
Crystal models of (a) Nb_12_Mo_4_H, (b) a tetrahedral interstitial site (TIS), and (c) an octahedral interstitial site (OIS). The red and black balls represent Nb and Mo atoms, respectively. The small blue balls represent various sites of H atoms.

Accordingly, we built supercell models of basic defect structures in which a H atom was placed at the tetrahedral interstitial site (TIS) and another at the octahedral interstitial site (OIS). A unit cell of 2 × 2 × 2 (16 atoms) with a body-centered cubic (BCC) structure^23^ was selected for pure Nb, and a Mo atom was introduced to replace the Nb atom—a series of structures with the composition Nb_16−*x*_Mo_*x*_ was thus obtained. One H atom was then added at the TIS and one at the OIS of BCC Nb and Nb_16−*x*_Mo_*x*_. Fig. 1 shows the schematic illustrations of Nb_16−*x*_Mo_*x*_H (*x* = 4) with TIS and OIS.

To probe the diffusion properties of hydrogen in the bulk of the Nb–Mo alloy, the climbing image nudged elastic band (CI-NEB) method^24^ was used to determine the diffusion barriers between the initial and final positions. Four images were taken and all the images were relaxed until the maximum force on each atom was less than 0.01 eV Å^−1^ and the other computational parameters were the same as the above.

The diffusion coefficient (*D*) is also an important index that determines the diffusion velocity of H. According to the Arrhenius diffusion equation, *D* can be expressed by *D* = *D*_0_ exp(−*E*_a_/*kT*), where the *D*_0_, *E*_a_, *k*, and *T* are the pre-exponential factor, diffusion energy barrier, the Boltzmann constant, and the absolute temperature, respectively. For a metal with a cubic structure, *D*_0_ can be expressed as 
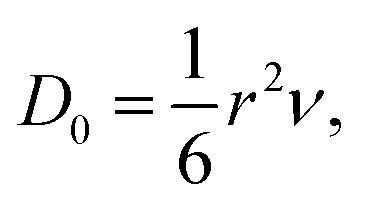
 where *r* and *ν* are the jump distance and the vibration frequency, respectively. We calculated *ν* according to Zener and Wert's theory,^25^ which is approximately expressed by 
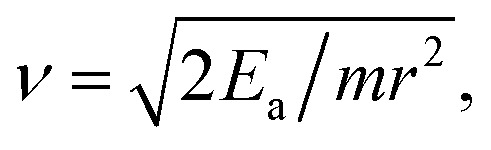
 where *m* is the mass of the impurity atom. As it is already known that the mass of the H atom is 1.67 × 10^−27^ kg, the jumping distance of the TIS H in Nb was set as 
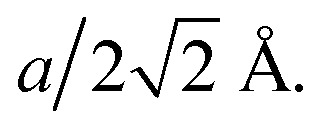


## 3. Results and discussion

### 3.1. Structure stability of Nb_16−*x*_Mo_*x*_H phases

**Fig. 2:**
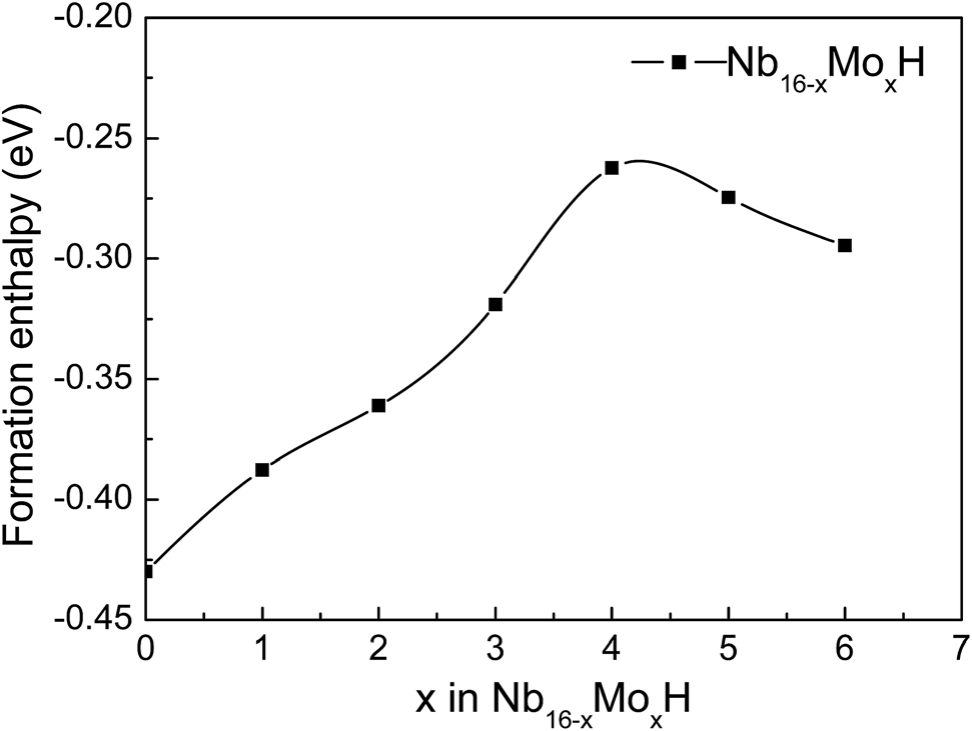
Formation enthalpies of Nb_16−*x*_Mo_*x*_H (*x* = 0, 1, 2, 3, 4, 5, 6) phases.

In order to investigate the effect of the amount of alloying Mo on the stability of the Nb_16_H phase, the heats of formation (Δ*H*_f_) of various Nb_16−*x*_Mo_*x*_H (*x* = 0, 1, 2, 3, 4, 5, and 6) phases were calculated from the following equations:1
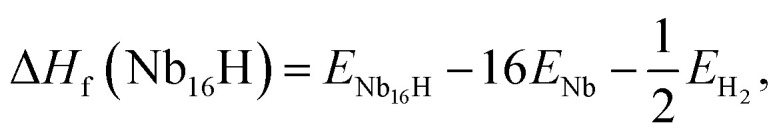
2
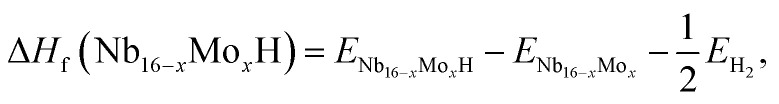
where *E*_Nb_16_H_, *E*_Nb_16−*x*_Mo_*x*_H_, *E*_Nb_16−*x*_Mo_*x*__, *E*_Nb_ and *E*_H_2__ are the total energies of Nb_16_H, Nb_16−*x*_Mo_*x*_H, Nb_16−*x*_Mo_*x*_, BCC Nb (ground state), and a H_2_ molecule, respectively. The obtained Δ*H*_f_ values of the Nb_16−*x*_Mo_*x*_H phases are presented in Fig. 2 and Table 1.

**Table 1:** Calculated lattice constants (*a*), elastic constants (*C*_*ij*_), bulk modulus (*B*), shear modulus (*G*), and Young's modulus (*E*) of Nb_16_, Nb_16_H, and Nb_12_Mo_4_H phases

Phase	Lattice constant, *a* (Å)	Mechanical property (GPa)											
		*C*_11_	*C*_12_	*C*_13_	*C*_23_	*C*_22_	*C*_33_	*C*_44_	*C*_55_	*C*_66_	*B*	*G*	*E*
Nb_16_	6.60	244	139.3					13.7			174.2	24.3	69.7
Nb_16_H (TIS)	6.63	245	137.8	138			247.2	25.4		26.5	173.8	34.8	98
Nb_16_H (OIS)	6.58	242	148.3	128			274.4	14.5		13.7	174.1	26	74.4
Nb_12_Mo_4_H (TIS)	6.53	311	137.4	143	139.2	278.9	317.6	31.6	39	39.7	194.9	50.6	139.7

Several characteristics can be derived from Fig. 2 and Table 1. First, all of the Nb_16−*x*_Mo_*x*_H phases are energetically favorable, with negative Δ*H*_f_ values. The absolute values of Δ*H*_f_ are very small (less than 0.2 eV), indicating that the Nb_16−*x*_Mo_*x*_H phases can be stable. Second, the absolute value of the formation enthalpy of Nb_16−*x*_Mo_*x*_H increases as the amount of alloying Mo increases from *x* = 0 to *x* = 4; it then decreases when *x* is above 4. The Nb_12_Mo_4_H phase has the highest absolute value of Δ*H*_f_, which is favorable for the dehydrogenation of the Nb_16−*x*_Mo_*x*_H phase. Therefore, Nb_12_Mo_4_H was selected for further study. Third, the calculated formation enthalpy absolute values of Nb_16_H (TIS) and Nb_16_H (OIS) are 0.43 and 0.09 eV, respectively. Nb_16_H (TIS) has a clearly higher absolute value of 0.43 eV, which indicates that the TIS is thermodynamically more possible than the OIS for H in BCC Nb. Such results are in excellent agreement with similar experimental observations^26–28^ and theoretical work^14^ reported in the literature. Since the addition of Mo has no effect on the preferred location of H, and TIS is the predominant location of H in both Nb_16_H and Nb_12_Mo_4_H phases, only the Nb_12_Mo_4_H (TIS) phase was selected for further investigation of its mechanical properties. In addition, the Nb_12_Mo_4_H (TIS) phase is energetically less favorable, with higher Δ*H*_f_ value than the corresponding Nb_16_H (TIS), suggesting that the addition of Mo would decrease the solubility of H in the TIS of Nb. A possible explanation has been offered by Yukawa *et al.*: the higher Δ*H*_f_ basically induces an decrease in H solubility and hydrogen embrittlement.^7,13,18^

After the series of calculations for the supercell models, the lattice constants (*a*) of various Nb_16_H and Nb_12_Mo_4_H phases were obtained; the results are listed in Table 1. The calculated lattice constants of pure Nb, Nb_16_H (TIS and OIS) and Nb_12_Mo_4_H (TIS) are 6.60, 6.63, 6.58, and 6.53 Å, respectively. The values of pure Nb and Nb_16_H (TIS) match well with the corresponding experimental unit cell values of 6.61 and 6.63 Å.^26–28^ In addition, Mo has a smaller atomic radius than Nb, which may lead to a slight decrease in the lattice constant with the addition of Mo to the Nb_16_H phase.

### 3.2. Mechanical properties of Nb_16_H and Nb_12_Mo_4_H phases

To find out the effect of Mo alloying on the mechanical properties of Nb hydride, the elastic constants of Nb_16_H (TIS and OIS), Nb_12_Mo_4_H (TIS), and pure Nb were calculated for comparison. The specified elastic constant was obtained by analyzing the difference between the total energy of the original cell and that of the deformed cell under a series of small strains. For the BCC crystal of Nb, there are three independent components of elastic constants: *C*_11_, *C*_12_, and *C*_44_. Nb_16_H has tetragonal symmetry and possesses three more components: *C*_13_, *C*_33_, and *C*_66_. Mo doping reduces the symmetry to orthorhombic and thus adds two components: *C*_22_ and *C*_23_.^29,30^ The calculated elastic constants are listed in Table 1.

According to the equations of elastic moduli and the criteria for mechanical stability, the mechanical stability is defined by the following restrictions for a tetragonal crystal:^31,32^3
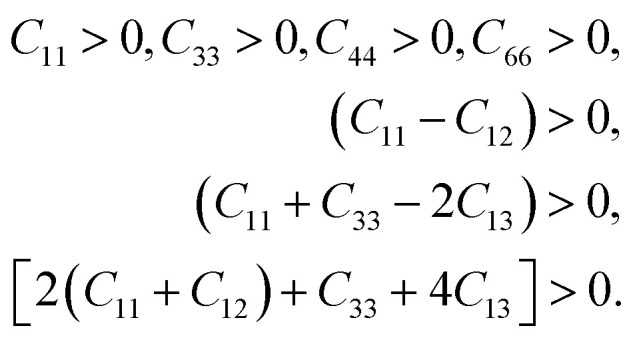


For an orthorhombic crystal, the criteria of mechanical stability are given by4
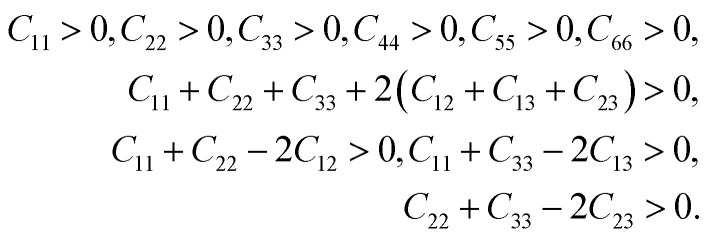


The results of elastic constants, *C*_*ij*_, indicate that both Nb_16_H and Nb_12_Mo_4_H phases meet the criteria for mechanical stability.

The obtained elastic constants were then used to calculate the bulk modulus (*B*) and shear modulus (*G*) from the Voigt–Reuss–Hill approximations.^33,34^ The Young's modulus (*E*) is determined using the equation *E* = 9*BG*/(3*B* + *G*).^31^ The values of *B*, *G*, and *E* of the Nb_12_Mo_4_H (TIS) phase are larger than those of the Nb_16_H (TIS and OIS) phase, indicating that Mo alloying improves the mechanical properties of the Nb_16_H phase, which possibly enhances the resistance against hydrogen embrittlement.

### 3.3. Electronic properties of Nb_16_H (TIS) and Nb_12_Mo_4_H (TIS)

**Fig. 3:**
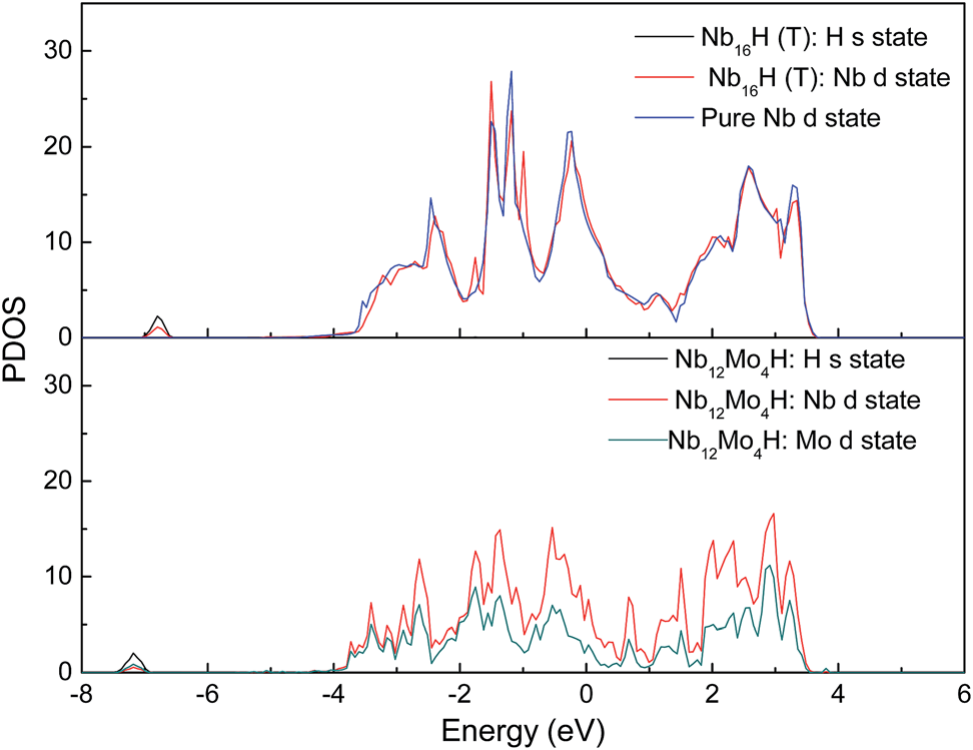
Comparison of the projected density of states (PDOS) of Nb_16_H (TIS) and Nb_12_Mo_4_H (TIS) phases.

**Fig. 4:**
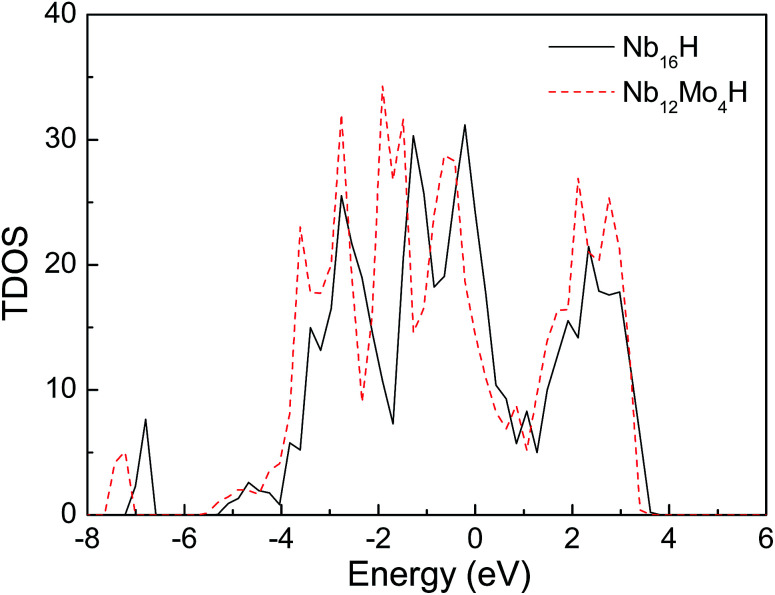
Comparison of total density of states of Nb_16_H (TIS) and Nb_12_Mo_4_H (TIS) phases.

Fig. 3 displays the projected density of states (PDOS) of H atoms in the Nb_16_H (TIS) phase and the Nb_12_Mo_4_H (TIS) phase. It can be seen that there is an obvious difference between the PDOS of Nb_16_H (TIS) and that of Nb_12_Mo_4_H (TIS). The PDOS peaks at about −6.8 eV are primarily Nb d states in Nb_16_H (TIS), whereas the PDOS peaks of Nb_12_Mo_4_H (TIS) are dominated by the overlapping H s, Nb d, and Mo d states, indicating that the H atoms penetrated the alloy and bonded with the Nb atoms. After doping with Mo atoms, the hybridizations peaks between the H s state and the Nb d state are smaller and shifted toward lower energy. Fig. 4 shows the total density of states (TDOS) of H atoms in the Nb_16_H (TIS) and Nb_12_Mo_4_H phases. Compared with the TDOS of the Nb_16_H (TIS) phase, the TDOS of the Nb_12_Mo_4_H (TIS) below the Fermi level (*E*_F_) are shifted leftward to the positions with slightly higher binding energies. These features of electronic structures suggest that the Nb_12_Mo_4_H (TIS) phase should have a stronger chemical bonding than Nb_16_H (TIS), which is beneficial to hydrogen permeation and should help one understand the improved mechanical properties (*B*, *E*, and *G*) shown in Table 1. In addition, the PDOS peaks are energetically degenerate with the addition of Mo in all regions, which indicate that the Nb–H hybridization is remarkable and favorable for improving the stability of the corresponding system.

### 3.4. Diffusion and permeation of H in Nb and Nb_12_Mo_4_

Next, we investigated the effect of Mo addition on the diffusion of hydrogen in the Nb_16_H and Nb_12_Mo_4_H phases. The CI-NEB method was used to determine the minimum-energy path and the corresponding energy barrier for the process of H diffusion in the Nb and Nb_12_Mo_4_ phases.^35–37^ For both Nb and Nb_12_Mo_4_, there are three possible paths of H diffusion between TIS and OIS, namely, from TIS to TIS, from TIS to OIS, and from OIS to OIS. Since the diffusion path from OIS to OIS cannot not be realized because the TIS is just located along the path, it was excluded in our study.^38–40^

**Fig. 5:**
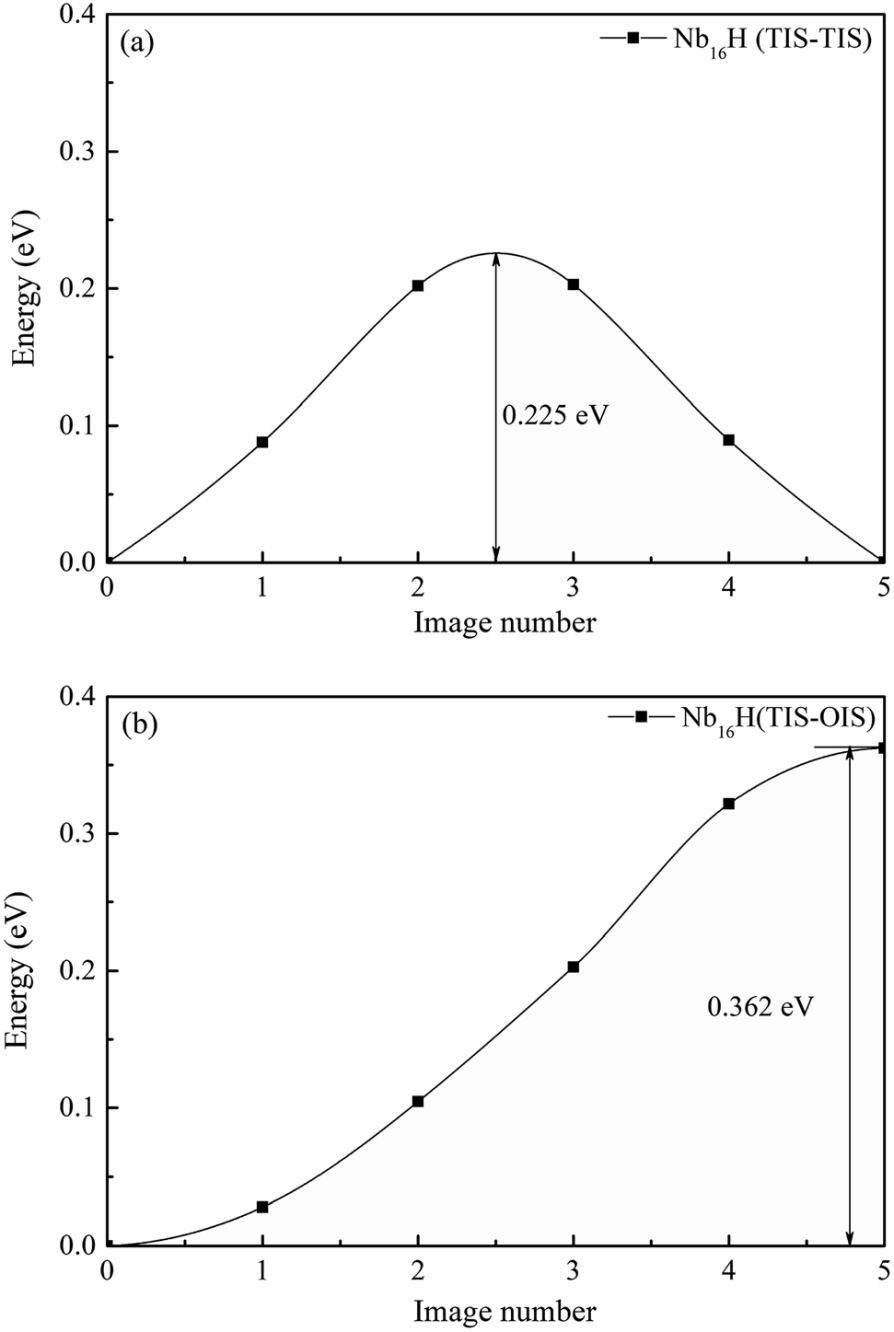
Migration barrier of H diffusion (a) from TIS to TIS and (b) from TIS to OIS in the Nb_16_H phase.

**Fig. 6:**
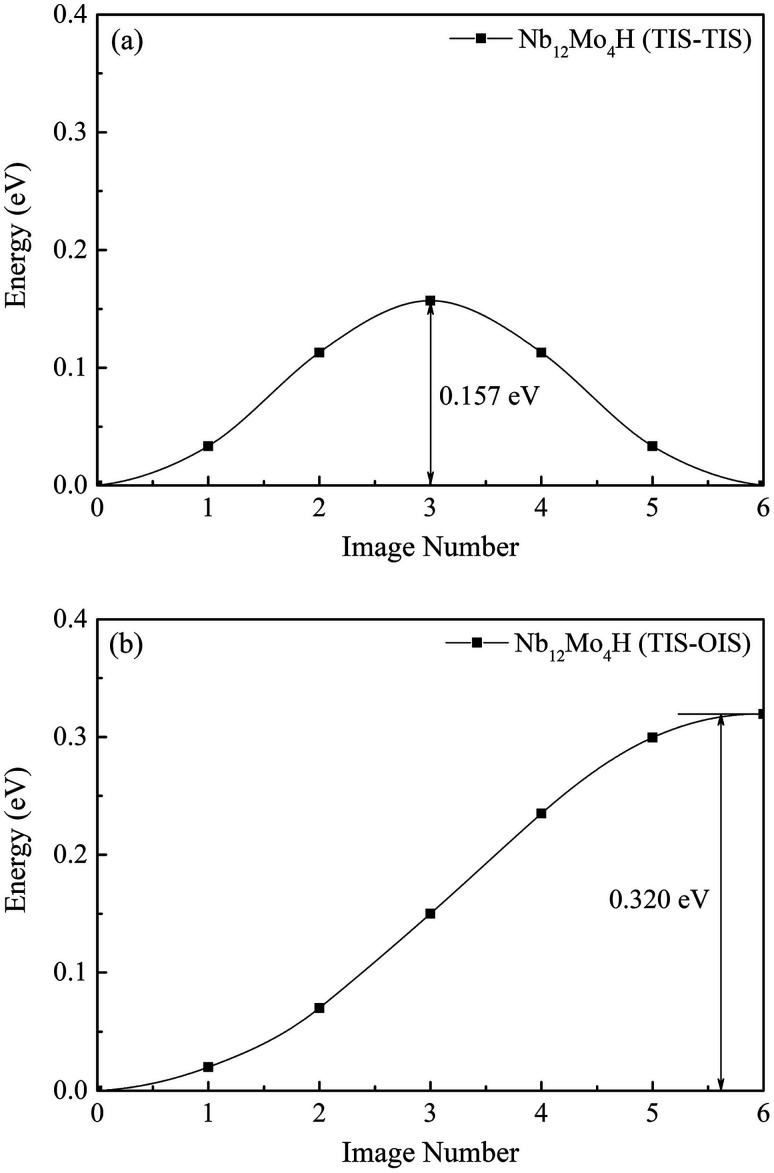
Migration barrier of H diffusion (a) from TIS to TIS and (b) from TIS to OIS in the Nb_12_Mo_4_H phase.

We first investigated the pathways of H in the bulk of pure Nb. Generally, there are two pathways for H to diffuse in a BCC lattice, *i.e.*, TIS → TIS and TIS → OIS. It can be clearly seen that the energy barrier from TIS to TIS in Nb is 0.225 eV, which is much lower than the corresponding value of 0.362 eV from TIS to OIS, suggesting that the diffusion path of H in bulk Nb should be mainly from TIS to TIS instead of TIS to OIS. For H diffusion in the Nb_12_Mo_4_ alloy, it can be clearly seen in Fig. 5 and 6 that the energy barrier from TIS to TIS (0.157 eV) is smaller than the corresponding value in Nb, and a similar observation can be seen from TIS to OIS. The above comparison demonstrates that the addition of Mo in Nb can make H diffusion easier with a smaller energy barrier.

The H-diffusion energy barrier calculated for pure Nb and the Nb_12_Mo_4_ alloy is 0.225 and 0.157 eV, respectively. The vibration frequency of pure Nb and the Nb_12_Mo_4_ alloy is 2.686 × 10^13^ s^−1^ and 2.345 × 10^13^ s^−1^, respectively. According to the Arrhenius diffusion equation, the calculated diffusion coefficient is 8.87 × 10^−7^ cm^2^ s^−1^ for pure Nb and 5.65 × 10^−6^ cm^2^ s^−1^ for the Nb_12_Mo_4_ phase at the standard room temperature of 300 K. From the above analysis, it can be deduced that the addition of Mo should have an important effect on the diffusion of H in Nb, *i.e.*, H diffusion in the Nb_12_Mo_4_ phase should become energetically more favorable when the energy barrier is lower. These characteristics would therefore bring about an increase in the H-diffusion coefficient and an improvement in H permeability. In other words, the addition of Mo could lower the diffusion barrier of H, which would fundamentally lead to higher H diffusion and high H permeability in the Nb_12_Mo_4_H phase.

## 4. Conclusions

We used first-principles calculations and the CI-NEB method to perform a comprehensive study on the effects of Mo on the structural stability and mechanical properties of the Nb_16_H phase and the diffusion of hydrogen through the alloy. The calculations revealed that the Nb_12_Mo_4_H phase is the most thermodynamically stable structure with a low Δ*H*_f_ of about −0.26 eV and a high elastic modulus. The diffusion paths of H in both Nb and Nb_12_Mo_4_ phases should be mainly from TIS to TIS. The calculated H-diffusion energy barrier and the diffusion coefficient are 0.153 eV and 5.65 × 10^−6^ cm^2^ s^−1^ (at 300 K), respectively. The lower energy barrier and higher diffusion coefficient of the Nb_12_Mo_4_ phase imply that the addition of a suitable amount of Mo could improve hydrogen permeation in Nb metal.

## Conflicts of interest

None.

## Supplementary Material
